# Diagnostic criteria and endoscopic and histological findings of autoimmune gastritis in Japan

**DOI:** 10.1007/s00535-022-01954-9

**Published:** 2023-03-01

**Authors:** Tomoari Kamada, Hidenobu Watanabe, Takahisa Furuta, Shuichi Terao, Yasuhiko Maruyama, Hiroshi Kawachi, Ryoji Kushima, Tsutomu Chiba, Ken Haruma

**Affiliations:** 1 Department of Health Care Medicine, Kawasaki Medical School General Medical Center, 2-6-1, Nakasange, Kita-Ku, Okayama, 700-8505 Japan; 2 Pathology and Cytology Laboratories Japan, Tokyo, Japan; 3 Center for Clinical Research, Hamamatsu University School of Medicine, Hamamatsu, Japan; 4 Department of Gastroenterology, Kakogawa Central City Hospital, Kakogawa, Japan; 5 Department of Gastroenterology, Fujieda Municipal General Hospital, Fujieda, Japan; 6 Department of Pathology, Cancer Institute Hospital, Japanese Foundation for Cancer Research, Tokyo, Japan; 7 Department of Pathology, Shiga University of Medical Science, Otsu, Japan; 8 Kansai Electric Power Hospital, Osaka, Japan; 9 Department of General Internal Medicine 2, Kawasaki Medical School, Kurashiki, Japan

**Keywords:** Autoimmune gastritis, Type A gastritis, Diagnostic criteria, Early-stage AIG, Corpus-predominant atrophy

## Abstract

The Japanese diagnostic criteria for autoimmune gastritis (AIG) were established by the “Study Group on the establishment of diagnostic criteria for type A gastritis,” which is related to a workshop associated with the Japan Gastroenterological Endoscopy Society (JGES) and the Committee of AIG Research Group (CARP). The criteria were set as follows: the cases of confirmed diagnosis are patients in whom either the endoscopic or histological findings, or both, meet the requirements for AIG and who are confirmed to be positive for gastric autoantibodies (either anti-parietal cell or anti-intrinsic factor antibodies, or both). The presentation of endoscopic findings of early-stage AIG in the diagnostic criteria was withheld owing to the need for further accumulation and characterization of endoscopic clinical data. Therefore, diagnosis of early-stage AIG only requires histological confirmation and gastric autoantibody positivity. Suspected cases are patients in whom either the endoscopic or histological findings, or both, meet only the requirements for AIG. Histological findings only meet the requirements for early stage. AIG has been underdiagnosed in the past, but our study group’s newly proposed diagnostic criteria will enable a more accurate and early diagnosis of AIG. The criteria can be used to stratify patients into various high-risk groups for gastric tumors and pernicious anemia. They would allow the establishment of an appropriate surveillance system in the coming years. Nevertheless, issues such as establishing the endoscopic findings of early-stage AIG and obtaining Japanese insurance coverage for gastric autoantibody tests require attention.

## Introduction

Autoimmune gastritis (AIG, type A gastritis) is a unique type of gastritis in which parietal cells are destroyed by autoimmune mechanisms, resulting in the production of autoantibodies (anti-parietal cell antibodies: PCA) against proton pumps (H^+^/K^+^ ATPase). In 1973, Strickland and Mackay [1] reported two types of chronic atrophic gastritis—types A and B, with the former regarded as the end stage of AIG.

Until recently, AIG has been considered a rare disease in Japan; however, its frequency has steadily risen [2–4]. Possible factors contributing to the increase in AIG include the promotion of endoscopic screening; group D (negative for *H. pylori* and positive for pepsinogen [PG] [5]) findings in gastric cancer risk stratification tests; evaluation for macrocytic anemia [6], iron deficiency anemia, thyroid disease [7–9], and gastric cancer [10–16]; close examination of gastric neuroendocrine tumors (NETs) [17–24]; and the presence of AIG in a subset of repeat cases of *H. pylori* eradication by ^13^C-urea breath test (UBT) [25]. Terao et al. [26] retrospectively reviewed the clinical and endoscopic characteristics of 245 AIG cases collected from 11 institutions in Japan and showed that the best diagnostic indicators for AIG were endoscopic findings (31.2%), followed by macrocytic anemia (16.1%), and repeated *H. pylori* eradication due to ^13^C-UBT false positivity (15.1%).

A characteristic endoscopic finding of AIG is corpus-dominant advanced atrophy [26–31]. In addition, sticky adherent dense mucus and remnant oxyntic mucosa may be observed in the corpus [26–31]. Moreover, magnified endoscopic findings [32–34], such as white globe appearance (WGA) [35, 36] and cast-off skin appearance (CSA) [36], have been reported, with few reports of early-stage AIG in which reverse atrophy has not fully developed [30, 37–41].

Several factors have clinical significance in the diagnosis of AIG. First, vitamin B12 deficiency increases the risk of pernicious anemia and neurological diseases such as subacute combined degeneration of the spinal cord [42, 43], peripheral neuropathy, and dementia. Second, hyposecretion of gastric acid can lead to iron deficiency anemia. Third, a high risk of gastric cancer and gastric NETs increases the risk of developing AIG. Finally, autoimmune diseases such as Hashimoto’s thyroiditis are often associated with other organs (polyglandular autoimmune syndrome IIIb) [44].

However, currently, no clear diagnostic criteria for AIG have been established. Thus, a comprehensive diagnosis is made from findings such as atrophic endoscopic findings in the gastric body to the fundus, presence of gastric autoantibodies, high serum gastrin levels, and characteristic histological findings (destruction/disappearance of parietal cells, pseudopyloric gland or intestinal metaplasia, enterochromaffin-like [ECL] cell hyperplasia, and gastrin cell hyperplasia).

In this article, we propose the diagnostic criteria for AIG in Japan, including its endoscopic and histologic characteristics.

## Background to the creation of the Japanese diagnostic criteria for AIG

Numerous cases should be examined to create the diagnostic criteria for AIG. In 2015, the Committee of AIG Research Group (CARP) was established, which centered on approximately ten facilities in Japan that were actively conducting clinical research on AIG. An achievement of this meeting was that the results of a multicenter study involving a clinicopathological examination of 245 AIG cases were reported at the American College of Gastroenterology in 2017 (Digestive Disease Week) and published in Digestive Endoscopy in 2020 [27].

“Study Group on the establishment of diagnostic criteria for type A gastritis”, an affiliated study group of the JGES, was established in 2019. By May 2021, three study groups were held (published in 2020), and in May 2022, the diagnostic criteria were published as a report of results [31]. The endoscopic findings in these diagnostic criteria were established based on discussions at the CARP, the results of the multicenter study [27], and presentations at affiliated study groups. The histological findings were selected based on evidence such as international diagnostic criteria [45–62] and presentations and papers by the affiliated study groups [63, 64].

## Outline of the diagnostic criteria for AIG

In Western countries, AIG diagnosis is based on the characteristic histopathological findings, the presence of PCA, and/or anti-intrinsic factor antibody (IFA) tests [49–54], since the main purpose of endoscopy is to collect gastric mucosal tissues. However, this article discusses the diagnostic criteria for AIG, emphasizing endoscopic findings, histologic findings, and the presence of gastric autoantibodies. In Table 1, we showed the differences in diagnostic criteria for AIG between Japan and some other countries.

**Table 1:** Differences in diagnostic criteria for autoimmune gastritis between Japan and some other countries

Authors	Reference no. in the text	Country	Publication year	Journal	Histology	Gastric autoantibody	Endoscopic findings	Hypergastrinemia	Low serum cobalamin concentration	Pentagastrin-fast achlorhydria
Strickland et al	[1]	Australia	1973	Am J Dig Dis		◎				
Vargas et al	[49]	Spain	1995	Gut	◎	∘	∘	∘		∘
Centanni et al	[9]	Italy	1999	Arch Intern Med	◎			◎		◎
Torbenson et al	[45]	Baltimore	2002	Mod Pathol	◎					
Tozzoli et al	[69]	Italy	2010	Autoimmun Rev	◎			◎		
Rugge et al	[46]	Italy	2012	Aliment Pharmacol Ther	◎	◎				
Miceli et al	[50]	Italy	2012	Clin Gastroenterol Hepatol	◎	◎				
Pittman et al	[47]	Baltimore	2015	Am J Sur Pathol	◎					
Minalyan et al	[51]	USA	2017	Clin Exp Gastroenterol	◎					
Carabotti et al	[52]	Italy	2017	Medicine	◎	◎		◎		
Kalkan et al	[53]	Turkey	2017	Geriatr Gerontol Int	◎					
Zhang et al	[14]	China	2017	Scand J Gastroenterol	◎	◎			∘	
Massironi et al	[62]	Italy	2019	Autoimmun Rev	◎	∘		∘		
Kamada et al	This article	Japan	2023	J Gastroenterol	◎	◎	◎			

◎ main finding, ∘ secondary finding

Table 2 shows the diagnostic criteria for AIG. The cases of confirmed diagnosis were set as those that satisfied both of the following conditions: severe mucosal atrophy predominantly from the gastric body to the fundus based on endoscopic and/or histological findings, and positivity for gastric autoantibodies using PCA and/or IFA tests. The suspected cases were set as those that satisfied only the condition of severe mucosal atrophy predominantly from the gastric body to the fundus (Table 2). Serum gastrin and pepsinogen levels are useful for diagnosing AIG [65], but were not adopted in the diagnostic criteria because they are not specific findings.

**Table 2:** Diagnostic criteria for autoimmune gastritis

(Confirmed diagnosis)
(A) Either the endoscopic (*details) or histological findings (*details), or both, meet the requirements for autoimmune gastritis
(B) Gastric autoantibody positive [either anti-parietal cell (*details) or anti-intrinsic factor antibodies, or both] Those who meet both (A) and (B). (Histological findings and gastric autoantibody positivity only meet the requirements for early stage.)
(Suspicious diagnosis)
Those who meet only (A). (Histological findings only meet the requirements for early stage.)
(*Details)
Endoscopic findings < advanced stage >
(Main findings)
Severe mucosal atrophy predominantly from the gastric body to the fundus is observed (uniform mucosal blood vessels are visible)
(Secondary findings)
Sticky adherent dense mucus, remnant oxyntic mucosa, and hyperplastic polyps may be observed from the gastric body to the fundus The antral area is not always normally colored. Patchy redness, red streaks, and circular wrinkle-like patterns may serve as a reference Of the abovementioned items, the main findings are mandatory
Histological findings
Diagnosis is by dividing findings into three stages: early stage, advanced florid stage, and advanced end stage (Table 3)
Anti-parietal cell antibody
Tenfold or more is positive, although this may be changed in the future in consideration of false positives

Histological findings of early-stage AIG were included in this proposed diagnostic criteria, although its endoscopic findings were not presented owing to the need to accumulate further endoscopic clinical data [30, 37, 41]. Therefore, diagnosis of early-stage AIG requires histological confirmation and gastric autoantibody positivity. The suspected cases of early-stage AIG are those with no atrophy on endoscopy, only histological findings of early stage are noted, and gastric autoantibodies are absent (Table 2).

## Endoscopic findings of AIG

### Advanced stage

**Fig. 1:**
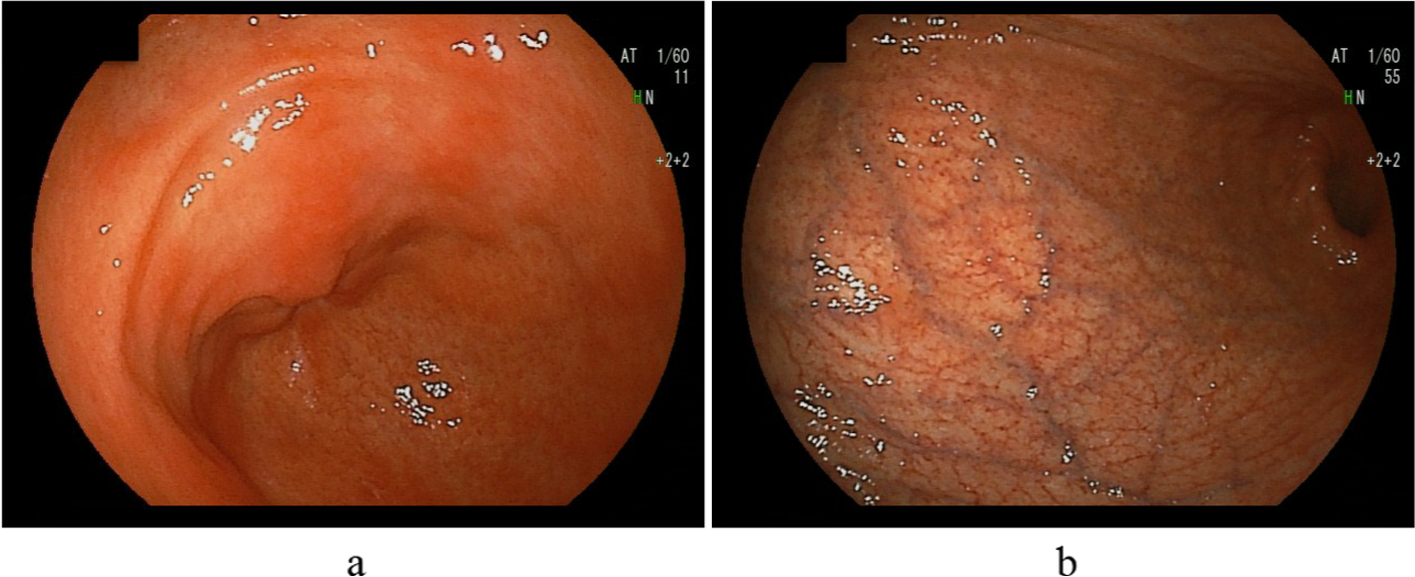
Endoscopic findings of advanced AIG (primary findings). Absent or mild antral atrophy, severe atrophy predominantly in the corpus (uniform mucosal blood vessels are visible), and no atrophic border. **a** Antral area. **b** Greater curvature of the corpus

AIG is characterized by corpus-dominant advanced atrophy, wherein uniform mucosal blood vessels are visible in the corpus [26–31]; this was used as the main diagnostic criterion (Fig. 1). A Japanese multicenter study [27] showed that the modified Kimura–Takemoto classification [66] O4 (O-P) was the most common gastric atrophy type (90.1%; 200/220), followed by O1–3 (5.9%; 13/221) among AIG patients. Most AIG cases do not show an atrophic border, although few cases show incomplete gastric body atrophy. For accurate diagnoses, it is essential to fully extend the greater curvature of the gastric body during endoscopy.

**Fig. 2:**
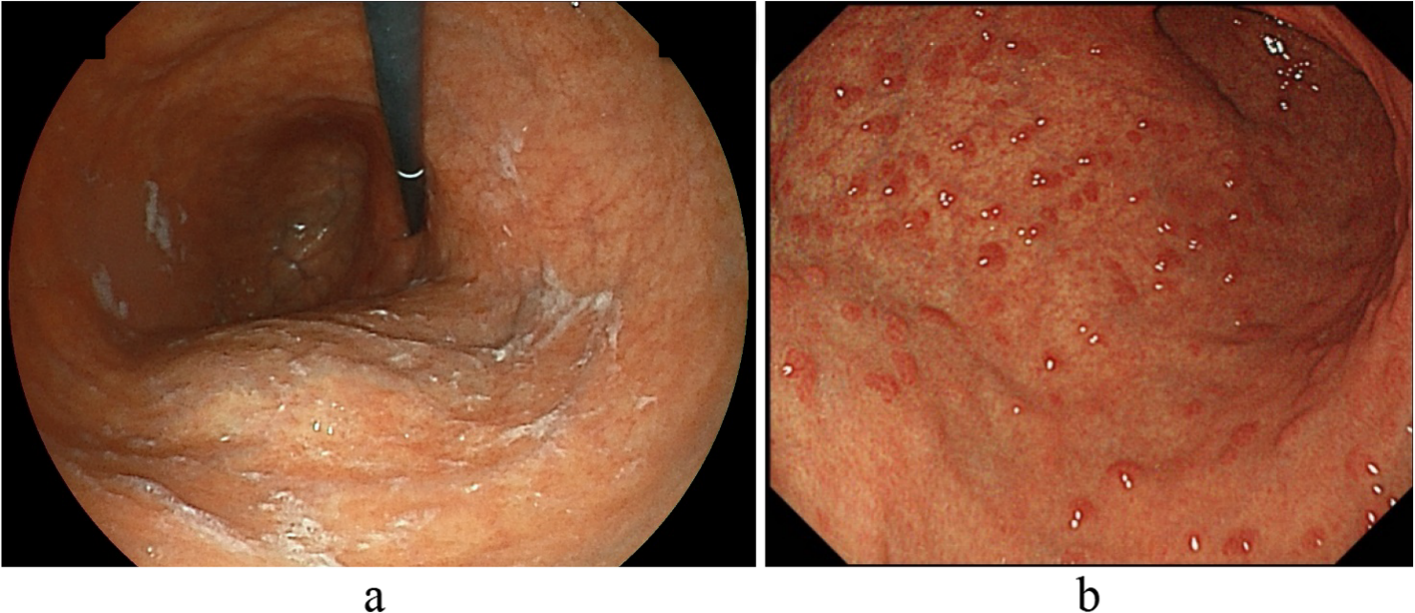
Endoscopic findings of advanced AIG (secondary findings). Sticky adherent dense mucus and residual fundic gland mucosa may be found in the corpus. **a** Sticky adherent dense mucus. **b** Remnant oxyntic mucosa (pseudopolyp-like)

In addition to corpus-dominant advanced atrophy, the sticky adherent dense mucus (Fig. 2a) and remnant oxyntic mucosa (Fig. 2b) are characteristic findings from the gastric body to the fundus, and hyperplastic polyps are useful for diagnosing AIG [26–31]. Sticky adherent dense mucus refers to pale yellow-to-white mucus formed in the fundus and upper part of the corpus due to decreased gastric juice secretion associated with severe mucosal atrophy. This adherent mucus cannot easily be removed by washing it with water. A Japanese multicenter study [27] reported adherent mucus in 32.4% of AIG patients. In addition, it has been reported that overgrowth of urease-positive bacteria other than *H. pylori* due to the sticky mucus associated with achlorhydria in AIG led to false-positive ^13^C-UBT results and misdiagnosis in patients who were refractory to eradication therapy [25].

Remnant oxyntic mucosa (oxyntic mucosa pseudopolyps [67]) refers to fundic gland mucosa that is preserved in a localized area during diffuse atrophy of the gastric mucosa. In a Japanese multicenter study [27], remnant oxyntic mucosa was observed in 31.5% of AIG cases. Its shape was classified into five types: flat localized (48.6%), pseudopolyp-like (22.9%), island shaped (18.6%), extensive (7.1%), and granular (2.9%).

Foveolar-type hyperplastic polyps are more frequently observed than conventional atrophic gastritis. Maruyama et al. [26] analyzed endoscopic findings in 20 AIG and non-AIG cases each and observed a significantly higher incidence of foveolar-type hyperplastic polyps in AIG cases (AIG: 50%; non-AIG: 15%).

**Fig. 3:**
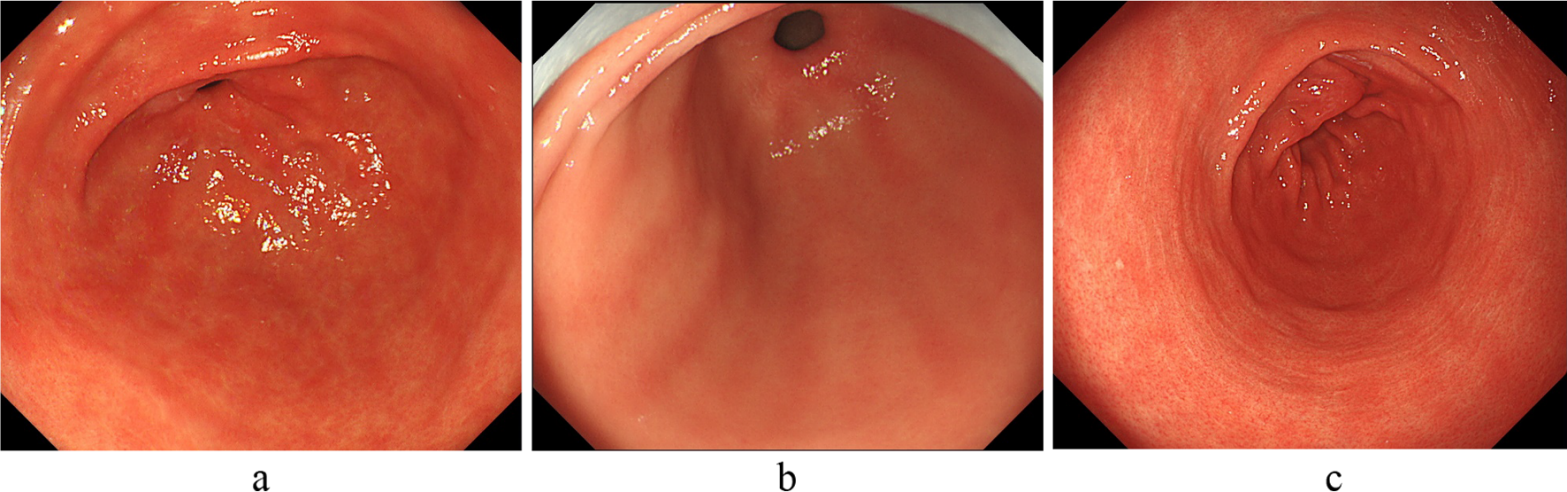
Mucosal findings of AIG (secondary findings) in the antral area. **a** Patchy redness. **b** Red streaks. **c** Circular wrinkle-like pattern

Although the antral area is generally considered to have no or mild atrophy and/or inflammation, there are cases in which the gastric mucosa is red or faded due to present or past *H. pylori* infection or bile reflux. Furthermore, patients with *H. pylori* infection also experience atrophy and/or inflammation; thus, the antral area is not necessarily a normal color. Additionally, patchy redness (Fig. 3a), red streaks (Fig. 3b), and circular wrinkle-like patterns (Fig. 3c) may also be helpful for AIG diagnosis [26–31].

### Early stage

Recently, early-stage AIG without severe mucosal atrophy has been reported [30, 37–41]. Kotera et al. [37] reported two cases of early-stage AIG in a 48-year-old man and a 70-year-old woman. In these patients, atrophic changes without intestinal metaplasia were predominant in the lesser curvature of the corpus, and the characteristic endoscopic AIG finding was pseudopolyp-like reddish nodules on the non-atrophic mucosa of the greater curvature of the corpus. Ayaki et al. [38] reported two cases of early-stage AIG in two women (35 and 40 years old). According to the authors, severe corporal atrophy was not complete, and swelling of the gastric mucosa in the corpus and a mosaic pattern in the fundus were observed. In addition, Kishino et al. [39] reported diffusely erythematous and edematous fundic gland mucosa and subepithelial capillary network expansion. Furthermore, Maruyama et al. [30] reported the findings of early-stage AIG as mildly edematous fundic gland mucosa without an observed regular arrangement of collecting venules (RAC) and diffuse spreading of enlarged gastric pits with a groove-like border. They reported that indigo-carmine spraying accentuated the mucosal pattern of the gastric pits, which were recognized as fine, salmon-roe-like nodular lesions.

**Fig. 4:**
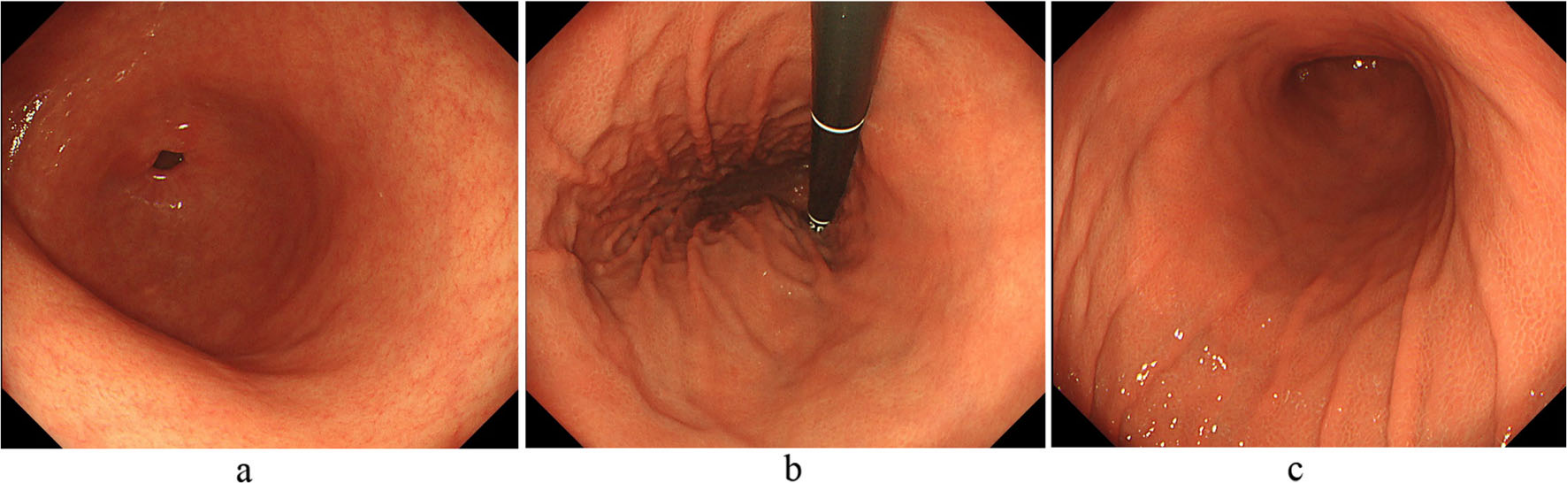
Endoscopic findings of early-stage AIG. Reddened and mildly edematous gastric pits are diffusely observed in the corporal mucosa without RAC despite the absence of *H. pylori* infection. The folds of the greater curvature of the corpus are preserved. **a** Greater curvature of the antral area. **b** Gastric body (observation looking upward). **c** Gastric body (observation looking downward)

Therefore, as Fig. 4 shows, early-stage AIG may be detected if reddened and mildly edematous gastric pits are diffusely observed in the corporal mucosa without RAC despite the absence of *H. pylori* infection. Particularly, suspecting AIG is crucial in complicated cases of thyroiditis. As described above, although common endoscopic findings of early-stage AIG are being documented, further accumulation and consideration of cases is desirable.

## Histological stage classification and histological features of AIG

Histological findings aid in classifying AIG into early stage, florid stage, and end stage, depending on the degree of progression of atrophy [48, 55–62]. Histological findings in our proposed diagnostic criteria are also divided into early stage, advanced florid stage, and advanced end stage, and their histological features are described (Table 3) [63, 64]. The definition of early-stage AIG concerning for histological findings differs greatly between Japan (Watanabe [63, 64]) and the West (Greenson [48]). Moreover, early-stage AIG in the West includes the advanced florid stage of AIG in the Japanese criteria.

**Table 3:** Histological stage classification and histological features of autoimmune gastritis

1 Early stage: may co-exist with the florid stage of AIG
(1) The ratio of the length of gastric pit to gastric gland in the fundic mucosa is almost normal, although the normal fundic gland structure (two-layered structure of parietal cell and mucous neck cell layer and chief cell layer) is obscured, and the entire gastric gland appears to be a parietal cell and mucous neck cell layer
Ratio of the length of the gastric pit and gastric gland: 1:2–4
Parietal cells: many remain in the gastric gland area, although they show degeneration (swelling = pseudohypertrophy), protrusion into the lumen, shedding, and slight decrease, accompanied by decreased proton pump staining and abnormal distribution within the cytoplasm
Chief cells (pepsinogen I positive and MUC6 negative): blurring and transformation to pyloric gland cells/mucous neck cells
Mucous neck cells (both pepsinogen I and MUC6 positive): distributed in the entire gastric glands, but marked increase at the bottom of gastric glands
Pyloric gland cells (pepsinogen I negative and MUC6 positive): (−) > (+), small number
Intestinal metaplasia (both CDX2- and CD10-positive small intestine type in the gastric body and fornix): ( −)
(2) Hyperplasia of ECL cells (chromogranin A-positive): ( −)–( +), intraductal, linear > small solid, small nodular
(3) Mild-to-moderate lymphocytic/plasma cell infiltration between the fundic glands. Lymphocytes (CD3^+^) are also present in the epithelium
(4) Gastrin cell hyperplasia: (+) – (−)
2 Advanced florid stage
(1) The gastric glands of the fundic mucosa are occupied by many-to-moderate numbers of mucous neck cell glands and pyloric glands. Prominent degenerated parietal cells and traces of parietal cell and mucous neck cell layer remain in some areas
Elongation of gastric pit (gastric gland pit) length and shortening of the gastric glands. Ratio of both lengths: 1: < 1 (~ 2)
Parietal cells: (−) > (+), few remaining parietal cells are degenerated, and proton pump staining is decreased/negative
Mucous neck cells: present in the lower half or bottom of the gastric glands or have disappeared
Pyloric gland cells: present in the upper part or entire length of gastric glands
Intestinal metaplasia (small intestine type): (−) to (+), mild
(2) ECL cell hyperplasia (intraductal / extraductal, linear > small solid, small nodular): (+)
(3) Gastrin cell hyperplasia: (+)
3 Advanced end stage: often co-exists with the florid stage of AIG
(1) Fundic mucosa is occupied by moderate-to-severe intestinal metaplasia and contains small amount of pyloric glands and mucous neck cell glands Alternatively, severe elongation of the gastric pit and small amount of gastric glands (pyloric glands > mucous neck cell glands) remain
(2) ECL cell hyperplasia (intraductal / extraductal, linear > small solid, small nodular): (+)
(3) Gastrin cell hyperplasia: (+)

To objectively recognize various cells, chromogranin A (synaptophysin is recommended if chromogranin A cannot be used) immunohistochemical staining can support the histological evaluation for ECL cells, H^+^/K^+^ ATPase staining for parietal cells, pepsinogen-I staining for chief cells, MUC6 staining for mucous neck cells, and gastrin staining for G cells. The recommended biopsy sites are the greater curvature of the pyloric antrum (2 cm proximal to the pyloric ring) and the greater curvature of the upper gastric body (however, when obtaining a biopsy specimen would impose a physical burden on the patient, such as in patients receiving antithrombotic therapy, using a single point in the greater curvature of the upper gastric body is acceptable). Furthermore, one biopsy of each of the gastric angles and middle lesser curvature of the gastric body is recommended to determine the presence or absence of atrophy in the gastric body and the presence or absence of *H. pylori* infection. Avoiding intestinal metaplasia as much as possible during a biopsy is important to accurately detect G and ECL cells [31, 63, 64].

**Fig. 5:**
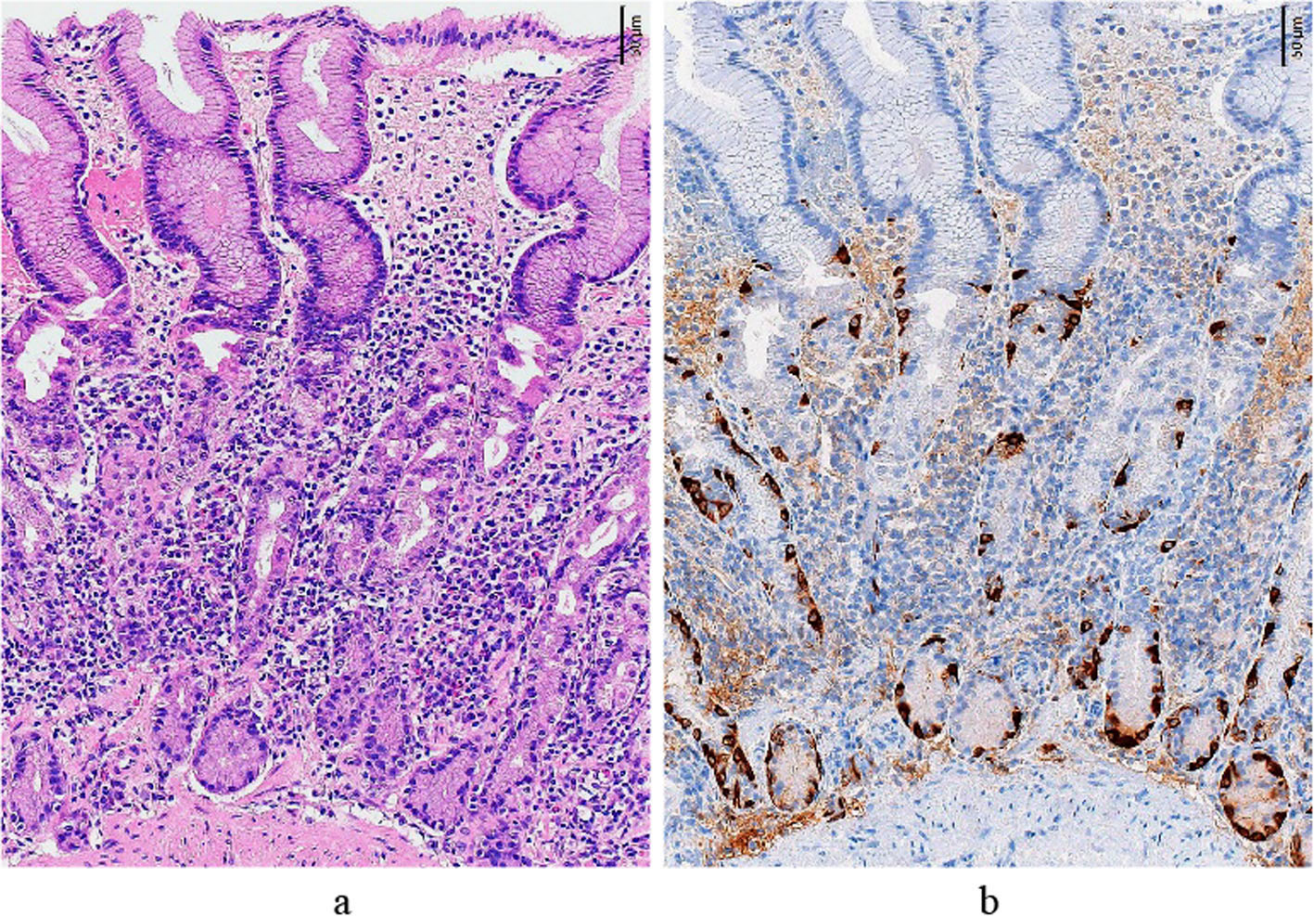
Histological features of early stage (greater curvature of the corpus). **a** The fundic gland mucosal structure that is almost similar to normal is preserved, although there is the elongation of the gastric pit (ratio of the gastric pit length to the gastric gland length: 1.0:1.8), and there is moderate chronic inflammatory cell infiltration between the fundic glands (stronger in the glandular area than in the gastric pit area). Numerous residual parietal cells exhibit pseudohypertrophy (degenerative swelling and cell protrusion). (HE staining). **b** Intraglandular hyperplasia of the ECL cells is observed (chromogranin A staining)

**Fig. 6:**
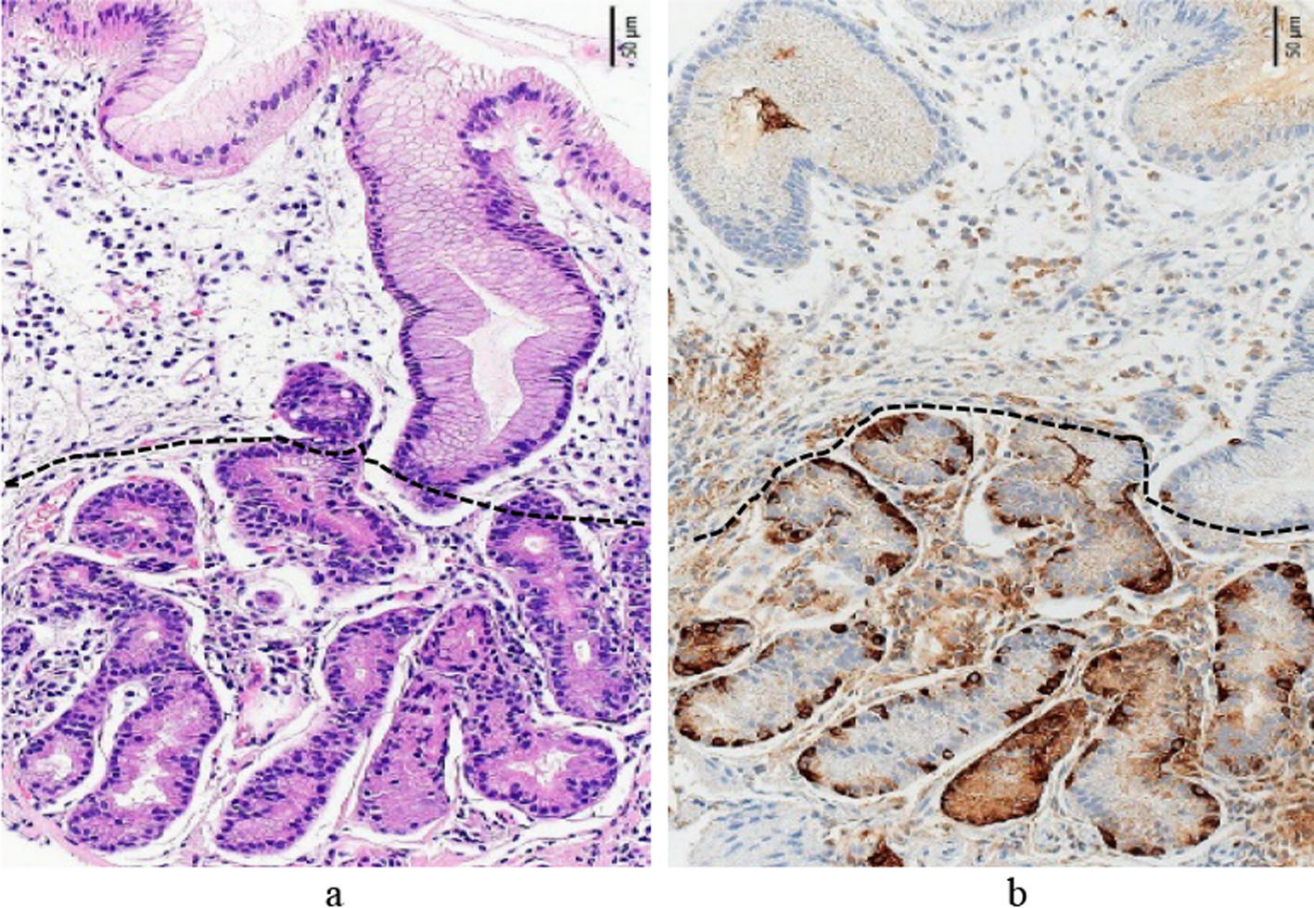
Histological features of advanced florid stage (greater curvature of the corpus). **a** The normal fundic gland mucosa structure has disappeared, the lengths of the gastric pit and glandular are 0.39 and 0.29 mm, respectively, (ratio: 1.0:0.7), and there is the elongation of the gastric pit and shortening of the gastric gland. The dashed line indicates the boundary between the gastric pit and gastric gland (HE staining). **b** Intraglandular linear hyperplasia of the ECL cells is observed (chromogranin A staining)

**Fig. 7:**
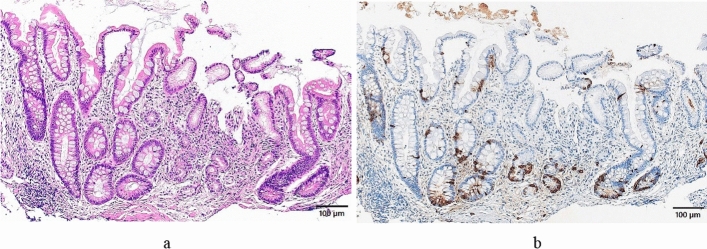
Histological features of advanced end stage (greater curvature of the corpus). **a** The normal fundic gland mucosa structure has disappeared, intestinal metaplasia has become severe, and a small amount of the pyloric gland mucosa remains at the center of this photo. The gastric pit length/the gastric gland duct length ratio is 0.34/0.08 mm (ratio: 4.2:1.0). (HE staining). **b** Intraglandular ECL cell hyperplasia has become clear. (chromogranin A staining)

- (1) Early stage (Fig. 5, Table 3)
  - Chief cells often transform to pyloric gland cells/mucous neck cells (pseudopyloric gland cells); thus, the normal two-layered structure of fundic glands, i.e., the parietal cell/ mucous neck cell layer and the chief cell layer are obscured, although the parietal and mucous neck cell layer is preserved. Many parietal cells remain, although swelling, intraluminal protrusion, and shedding (these changes in parietal cells are key findings for early stage) are observed. Hyperplasia of ECL is either absent or mild, and mild-to-moderate lymphocytic/plasma cell infiltration is observed between the gastric glands. Gastrin (G) cells in the pyloric gland mucosa also show mild hyperplasia in the early stage.
  - Early-stage AIG is diagnosed when the above-mentioned histological findings are observed. Lymphocytic infiltration/plasma cell infiltration in the stroma between the oxyntic glands and hyperplasia of G cells in the pyloric glands is also used as a diagnostic aid.
- (2) Advanced florid stage (Fig. 6, Table 3)
  - Gastric glands of the fundic mucosa are occupied by many-to-moderate numbers of mucous neck cell glands (pseudopyloric glands) and/or pyloric glands. Moreover, parietal cells are markedly decreased or absent if parietal cells are present, and marked degeneration would be noted. Additionally, elongation of the gastric pit and shortening of the fundic gland are observed, and hyperplasia of ECL cells is observed.
  - Marked decrease or disappearance of parietal cells is an important finding, and cases of this with ECL cell hyperplasia are diagnosed as advanced florid-stage AIG. G cell hyperplasia is also used as a diagnostic aid.
- (3) Advanced end stage (Fig. 7, Table 3)
  - This is a progression of the advanced florid stage. End stage findings may be additionally seen in some cases. The gastric glands are dominated by moderate-to-severe intestinal metaplasia and contain a small amount of pyloric and mucous neck glands (pseudopyloric glands). Elongation of the gastric pit is more advanced, only a small number of gastric glands remain, and hyperplasia of ECL cells can be observed. The diagnosis is the same as that of the advanced florid stage.

## Gastric autoantibodies

An autoimmune mechanism causes AIG; thus, gastric autoantibodies (PCA and IFA) are important diagnostic items, and their sensitivity and specificity are reported to be 81% and 90%, respectively, for PCA and 27% and 100%, respectively, for IFA [68]. However, the assessment of IFA is particularly expensive and is not covered by health insurance in Japan; thus, PCA, whose measurement cost is relatively inexpensive, is commonly used in many facilities. PCA concentration gradually increases to a peak value and then decreases as gastric mucosa alteration progresses [69, 70].

Notably, PCA is evaluated using a fluorescent antibody test in which the patient’s serum is diluted tenfold; a > tenfold result is considered positive, although a tenfold result may be a false positive. Therefore, the diagnostic criteria were set as “at least tenfold is positive, but there is a possibility of changes in the future in consideration of false positives.” Currently, the lack of insurance coverage in Japan for both gastric autoantibody tests is an issue, and it is hoped that the publication of our proposed diagnostic criteria will be a tool for soliciting insurance coverage for these tests.

## Conclusions

AIG diagnosis is significant for classifying patients into high-risk groups for gastric tumors, such as gastric cancer and NETs, and pernicious anemia, as well as for conducting regular surveillance. Our study group’s newly proposed diagnostic criteria in this article are expected to decrease the rate of underdiagnosis of AIG and promote the detection of early-stage AIG. Furthermore, the criteria can act as a framework for determining appropriate medical care. Urgent issues requiring resolution include establishing the endoscopic findings of early-stage AIG obtaining national health insurance coverage in Japan for gastric autoantibody tests.
